# Novel Therapeutic Savior for Osteosarcoma: The Endorsement of Ferroptosis

**DOI:** 10.3389/fonc.2022.746030

**Published:** 2022-03-24

**Authors:** Cheng Qiu, Tianyi Liu, Dan Luo, Dongyang Luan, Lin Cheng, Songgang Wang

**Affiliations:** ^1^ Department of Orthopaedic Surgery, Qilu Hospital, Cheeloo College of Medicine, Shandong University, Jinan, China; ^2^ Cheeloo College of Medicine, Shandong University, Jinan, China; ^3^ Department of Medical Oncology, National Cancer Center/National Clinical Research Center for Cancer/Cancer Hospital, Chinese Academy of Medical Sciences and Peking Union Medical College, Beijing, China; ^4^ College of Stomatology, Qingdao University, Qingdao, China; ^5^ Department of Emergency Medicine, Qilu Hospital of Shandong University, Jinan, China

**Keywords:** ferroptosis, osteosarcoma, drug resistance, GPX4, FSP1, GCH1, BH4, DHODH

## Abstract

Ferroptosis has recently been discovered as an iron-dependent and non-apoptotic regulated mechanism of cell death. The induction of ferroptosis in tumor cells improves tumor treatment, making it a current research hotspot. Mechanistically, it starts by lipid peroxidation, iron accumulation, reactive oxygen species (ROS) production, and glutathione deprivation, highlighting novel treatment opportunities for many tumors and neurodegenerative disorders. Several tumor cell lines are resistant to ferroptosis inducers, even when the ferroptosis key enzyme glutathione peroxidase 4 (GPX4) is blocked, indicating that other important elements are also involved in this process. Ferroptosis-suppressor-protein 1 (FSP1) was discovered to be one of these elements in addition to a few others such as ferroptotic gatekeepers like GTP cyclohydrolase 1 (GCH1) and dihydroorotate dehydrogenase (DHODH). Osteosarcoma is the most common primary malignant bone tumor observed most frequently in children and adolescents. Several studies demonstrated that ferroptosis plays a critical role in the treatment of osteosarcoma, in particular drug-resistant osteosarcoma cells. We outlined four primary regulators involved in ferroptosis in this article, reviewed previously published studies of ferroptosis in osteosarcoma to provide covert insights about osteosarcoma treatment, and highlighted several critical issues to point out future research possibilities.

## Introduction

Osteosarcoma is the most common primary malignant osseous tumor accounting for the largest proportion (60%) of orthopedic malignant tumors that commonly affect children and those younger than 20 years (1, 2). Distal femur and proximal tibia are the most common sites of osteosarcoma burst. However, the pathogenesis of osteosarcoma remains unclear, and it is considered to be related to the combination of genetic susceptibility, virus infection, ionizing radiation, and chemical toxins (3). Clinically, the main manifestations are swelling, pain, and dysfunction of adjacent joints, which can aggravate pain and affect patients’ sleep at night (4). Several studies demonstrate that 80% of patients with osteosarcoma have a local invasion or distant metastasis when diagnosed (5). The lung is the most common organ for tumor metastasis, accounting for 85% of cases, and 90% of patients with tumors die because of metastasis (6). Current therapeutic strategies for osteosarcoma include surgical resection, radiotherapy, chemotherapy, and immunotherapy with a five-year survival rate of 70% (7). The prognosis of osteosarcoma is still unoptimistic. Tumor cells are resistant to chemotherapeutic drugs. Drug resistance is a critical factor contributing to therapeutic failure and tumor recurrence. Therefore, extensive research on elucidating the mechanisms involved in osteosarcoma and identifying relative molecular targets as well as treatment methods is warranted.

Cell death is a fundamental biological process that pervasively takes place in all living organisms (8). Cancer cells evade quintessential immune surveillance-mediated cell death and then, due to overwhelming proliferation, eventually cause dysregulation in the body (9). The five widely accepted forms of cell death are necrosis, apoptosis, necroptosis, pyroptosis, and ferroptosis (8). Pressing engagement of unknown stimulation or toxic factors concerning the unit of life could trigger uncontrolled necrosis. Additional aforementioned types are accordingly ascribed to regulated cell death (RCD) (10). As a newly identified type of cell death, ferroptosis is a research hotspot, and it mechanistically occurs *via* lipid peroxidation, iron accumulation, reactive oxygen species (ROS) production, and glutathione deprivation, highlighting the novel treatment opportunities for drug-resistant tumors and neurodegenerative disorders (11). The gatekeepers of ferroptosis include glutathione peroxidase 4 (GPX4) (12), ferroptosis suppressor protein 1 (FSP1) (13, 14), GTP cyclohydrolase 1 (GCH1) (15, 16), and dihydroorotate dehydrogenase (DHODH) (17). Except for these four, other pathways such as acyl-CoA synthetase long-chain family member 4 (ACSL4), adenosine monophosphate activated protein kinase (AMPK)-ACC2, and NF2-YAP are also important in regulating ferroptosis; these regulate polyunsaturated fatty acid (PUFA) metabolism and cellular phospholipid composition (18–20). Lipoxygenases (ALOXs) and cytochrome P450 oxidoreductase (POR) have been known to affect ferroptosis by driving lipid peroxidation, which acts in ways opposite to GSH-GPX4, FSP1-CoQ10, GCH1-BH4, and DHODH (21). Looking at the current treatment options for osteosarcoma, we highlighted advances in ferroptosis and proposed several approaches to contribute to the basic study of osteosarcoma.

## Ferroptosis: A Novel Type of Regulated Necrosis

**Figure 1:**
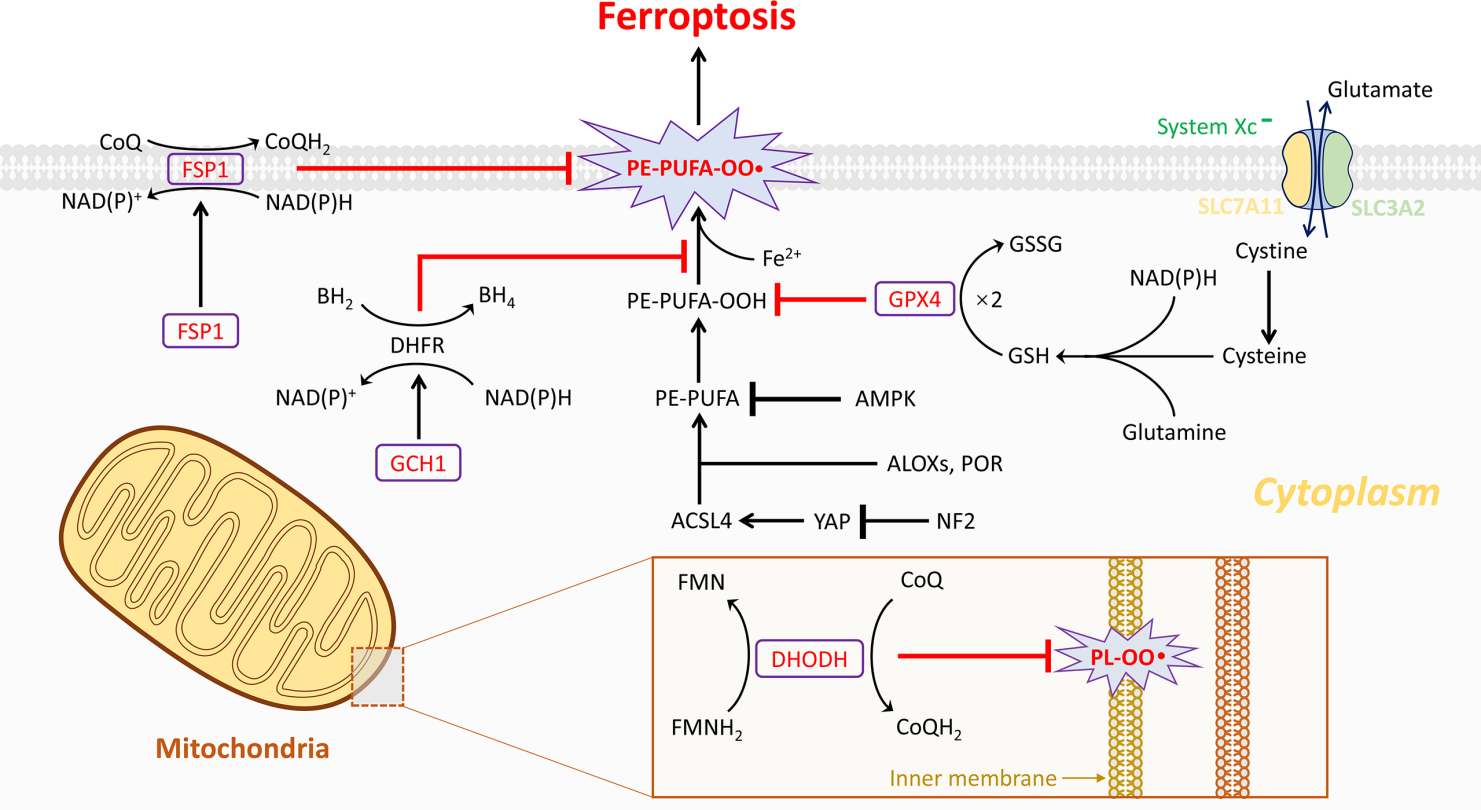
The four current known regulation systems in ferroptosis. Overall, lipid peroxidation in ferroptosis is under control of GPX4-, FSP1-, GCH1-, and DHODH-dependent systems. GPX4 is the most important gatekeeper for ferroptosis and bolstered through the sustainment of GSH and cystine transportation of system Xc^-^ activation. System Xc^-^ is composed of two essential subunits, SLC7A11 and SLC3A2. Generally, ferroptosis could be triggered by GPX4 inhibition directly or indirectly. Nonetheless, several cancer cell lines are resistant to GPX4 inhibition through activating additional regulation systems like FSP1/CoQ_10_ and GCH1/BH4 systems in the cytoplasm. These two independent manners play a critical role in mitigating cellular ferroptosis especially during loss of GPX4. However, the dysfunction of the three abovementioned systems is observed in organelles such as mitochondria. Notably, then the fourth antioxidant system DHODH-mediated ferroptosis protection in mitochondria is revealed. In the inner membrane of mitochondria, DHODH suppresses ferroptosis *via* the conversion of ubiquinone to ubiquinol that fights against oxidative damage on the phospholipid membrane. A total of four gatekeepers presumably serve as potential targets for the treatment of osteosarcomas. Except the four pathways, other pathways are also important in regulating ferroptosis, such as ACSL4, AMPK-ACC2, and NF2-YAP pathways, which have been known to affect ferroptosis by regulating PUFA metabolism and cellular phospholipid composition. Lipoxygenases (ALOXs) and POR have been known to affect ferroptosis by driving lipid peroxidation, which play opposite roles to GSH-GPX4, FSP1-CoQ10, GCH1-BH4, and DHODH.

This newly discovered type of regulated cell death, characterized by iron accumulation, glutathione (GSH) deprivation, and lethal lipid peroxidation, was first coined by Dixon et al. in 2012 (11). It was termed ferroptosis suggesting that this form of cell death was induced in an iron-dependent way, which made it morphologically, biochemically, and genetically distinct from the two well-known forms of cell death, namely, apoptosis and necroptosis (22). Hitherto, numerous efforts have been made to unravel this biological process and to establish a correlation between ferroptosis and diseases, including acute kidney injury (23), stroke (24), tuberculosis (25), ischemia/reperfusion injury (26), cardiomyopathy (27, 28), and spinal cord injury (29). Moreover, induction of ferroptosis could be considered as an effective strategy for treating tumor entities (30–35). Ferroptosis has become a research hotspot due to its extraordinary involvement in several untreatable diseases, and three major pathways have been identified (**Figure 1**).

Among them, the GPX4-GSH pathway is the most studied. With system Xc^-^ transferring glutamate and cystine, the synthesis of GSH depends on NAD(P)H thus reducing GPX4. The second pathway FSP1-CoQ_10_ (FSP1) could catalyze the reduction of CoQ_10_ through the oxidation of NAD(P)H to generate ubiquinol. In the GCH1-BH4 pathway, GCH1 could upregulate the level of BH4. All three abovementioned products can suppress ferroptosis and therefore have been discussed in more detail in this article (**Figure 1**).

## Ferroptosis Gatekeepers

### GPX4

Glutathione peroxidase (GPX), a classical catalysis family bolstered by GSH oxidation to reduce hydrogen peroxide (H_2_O_2_) to water, is composed of eight paramount members containing GPX1 to GPX8 (36). Selenocysteine is considered a unique component serving as the central active site for the first four enzymes and is also indispensable for the function of GPX4 (37). In coordination with GSH, GPX4 is important for regulating ferroptosis, as it can neutralize ROS and counter oxidation (**Figure 1**) (12, 37). In the GSH/GPX4 antioxidant system, upstream system X_c_^-^ comprises two subunits, solute carrier family 7 member 11 (SLC7A11) and SLC3A2, required for GSH/GPX4 biosynthesis, the antiporter transporting glutamate and cystine (38). The light chain SLC7A11 is demonstrated to be an effective therapeutic target for tumor treatment due to its unusual overexpression in many cancers. Generally, cellular GPX4 is prone to be inactivated by conventional class I ferroptosis inducers such as erastin, which specifically targets system X_c_^-^ to cause GSH depletion, indirectly resulting in iron-dependent lipid ROS peroxidation and subsequent ferroptosis (39, 40). However, class II ferroptosis inducers such as RSL3 could directly react with GPX4 to cause anti-oxidation disorders and elicit ferroptosis (12). Mechanistically, GPX4 mainly plays a specific role in counteracting ROS generated from the Fenton reaction, a classical chemical reaction involving ferrous iron and H_2_O_2_, to prevent ferroptosis (41).

### FSP1

Similar to GPX4, another promising protein named ferroptosis suppressor protein 1 (FSP1), earlier called apoptosis-inducing factor mitochondrial 2 (AIFM2), was discovered to mediate the ferroptosis protection pathway (13, 14). AIFM2 has been previously demonstrated to induce cell death in a caspase-independent manner, emerging as a potential therapeutic target for tumor treatment (42–44). Few reports have validated the key role of AIFM2 in inhibiting ferroptosis parallel to but independent of GPX4, whose name was then changed to FSP1. FSP1 is located on the human chromosome 10q22.1 and mediates p53-independent apoptosis (42). A majority of FSP1 protein adheres to the outer membrane of mitochondria, whereas the other part of FSP1 protein resides in the cytoplasm. Thus, the cytosolic FSP1 moves to the membrane when myristoylated to inhibit lipid peroxidation and arrest ferroptosis (13, 14). The N-myristoylation signal and flavoprotein oxidoreductase domain of FSP1 are essential to compete against ferroptosis. Moreover, FSP1 functions in collaboration with ubiquinone, also known as coenzyme Q10 (CoQ_10_). With a substantial utilization of NAD(P)H, FSP1 acting as an NADH-dependent CoQ_10_ oxidoreductase reduces ubiquinone to ubiquinol, which deals with oxidation through radical scavenging, thereby limiting lipid peroxidation and preventing the occurrence of ferroptosis (**Figure 1**). Overall, the deficiency of FSP1 and the restrained FSP1/CoQ_10_ signaling may play a critical role in causing cell death due to oxidative impingement.

### GCH1

Recently, the GTP cyclohydrolase 1/tetrahydrobiopterin (GCH1/BH4) pathway was verified to inhibit ferroptosis in a GPX4-independent manner (15, 16). At the cellular level, BH4 plays a critical role in the activities of various enzymes (45). Mechanically, BH4 counteracts ferroptosis by scavenging lipid peroxidation, providing strong protection during the anti-oxidation process, whereas GCH1 is a rate-limiting enzyme for the *de novo* biosynthesis of BH4 from guanosine triphosphate (GTP) (46). Likewise, ferroptosis is sensitized by simultaneously inhibiting GPX4 function and a recycling system governed by dihydrofolate reductase (DHFR), whereby BH4 regenerates from BH2 (**Figure 1**) (15, 16). Theoretically, BH4 confers robust defense against ferroptosis relying on the recycling shuttle loop of DHFR upon continuous replenishment by cellular hydrogen carriers. However, the underlying mechanisms of the GCH1/BH4 pathway are poorly understood in ferroptosis burst.

### DHODH

The FSP1-dependent CoQ_10_ reducing system, with antioxidant property, is termed as the endogenous antiferroptosis rudder (13, 14). The functions of FSP1 are strictly limited to cellular membranes according to current reports, and it was unclear whether mitochondrial membranes are subject to similar mechanisms. This question has now been satisfactorily answered by a recently published observation (17). Initially, in different types of cancer cells, lipid peroxidation drives the synthesis of pyrimidine bases in the presence of a specific enzyme dihydroorotate dehydrogenase (DHODH) located on mitochondria. DHODH catalyzes the conversion of dihydroorotate to orotate through an oxidative reaction by consuming ubiquinone and thereby generates ubiquinol to block ferroptosis, while this occurs independently in pyrimidine synthesis (**Figure 1**). Mitochondrial GPX4 possibly plays a redundant role in cell survival compared to DHODH; however, it repairs the lipid membrane damage (47). Potent DHODH inhibitors are sensitive to cancer cell lines with low GPX4 expression levels.

## Ferroptosis in Osteosarcoma

Recent studies have shown that ferroptosis is involved in tumor growth and malignancy (**Table 1**). Isani et al. first described ferroptosis-like cell death with iron-dependent and non-apoptotic characteristics in osteosarcoma cell line D-27 (55). After treatment with *Artemisinin annua* extract, D-27 cells showed cell death presented with ballooning phenotype instead of fragmented nuclei distinct from previous apoptosis as well as necrosis patterns and possessed aberrant iron levels. The study identified that ferroptosis sensitization could be induced by dampening STAT3/Nrf2/GPx4 signaling to enhance the sensitivity of osteosarcoma cells to cisplatin (54). This was the first study focused on drug-resistant osteosarcoma and revealed a novel approach to make osteosarcomas more sensitive to the drug by utilizing ferroptosis inducers or STAT3 inhibitors. The vital gatekeeper of ferroptosis, GPX4, was first reported to be involved in osteosarcoma. Moreover, phenethyl isothiocyanate (PEITC), an isothiocyanate that is effective against various cancers, was reported to stimulate osteosarcoma ferroptosis by impairing iron metabolism, redox balance, and GSH-iron-ROS regulation (52, 53). This form of osteosarcoma ferroptosis was observed to be controlled by the MAPK signaling pathway. Ultrasound-activatable doxorubicin (DOX)-Fe(VI)@HMS-HE-PEG (DFHHP) nanoparticles synergistically boosted the growth suppression of hypoxic osteosarcoma by inducing ferroptosis with notable GPX4 downregulation (51). Thereafter, three independent studies respectively demonstrated that the regulators EF24, KDM4A, and tirapazamine could mediate ferroptosis through GPX4 directly or indirectly (48–50). The overall picture of ferroptosis in osteosarcoma is incomplete, and further studies are warranted.

**Table 1:** Previous published studies regarding ferroptosis in osteosarcoma.

Year	Authors	Research object	Osteosarcoma cell lines	Target gate molecules	Major changes	Signaling pathways	Brief description
2021	Yihua Shi et al. (48),	Tirapazamine	HOS, 143B, U2os	SLC7A11	Iron accumulation, ROS production	/	Tirapazamine inhibits proliferation and migration of osteosarcoma cells under hypoxia by downregulation of SLC7A11 and GPX4
2021	Meng Chen et al. (49),	KDM4A	HOS, 143B	SLC7A11	GSH depletion, lipid peroxidation	/	KDM4A directly induces H3K9me3 demethylation to promote SLC7A11 transcription and dampen ferroptosis
2021	Haiyingjie Lin et al. (50),	EF24	U2os, Saos-2	GPX4	Lipid peroxidation, iron accumulation, ROS production	HO-1/GPX4	EF24 upregulates HMOX1 to suppress GPX4 expression to induce ferroptosis
2021	Jingke Fu et al. (51),	DFHHP nanomedicine	Saos-2	GPX4	GSH depletion, iron accumulation, ROS production, reoxygenation	/	Ultrasound-activatable DFHHP nanomedicine plays a synergistic role in boosting the growth suppression of hypoxic osteosarcoma by the induction of ferroptosis
2020	Huanhuan Lv et al. (52),	β-Phenethyl isothiocyanate	HOS, 143B, U2os, MG-63	GPX4	GSH depletion, lipid peroxidation, iron accumulation, ROS production	MAPK	β-Phenethyl isothiocyanate alters iron metabolism and disturbs the redox balance in osteosarcoma to induce cell death by activating the MAPK signaling pathway
2020	Huanhuan Lv et al. (53),	PEITC	K7M2	GPX4	GSH depletion, iron accumulation, ROS production	MAPK	PEITC induces ferroptosis in K7M2 osteosarcoma cells by activating the ROS-related MAPK signaling pathway
2019	Qiang Liu et al. (54),	STAT3	MG-63, Saos-2	GPX4	Lipid peroxidation, iron accumulation, ROS production	STAT3/Nrf2/GPX4	STAT3 inhibitors activate ferroptosis in osteosarcoma cells and increase sensitivity to cisplatin by impairing the STAT3/Nrf2/GPX4 signaling pathway
2019	Gloria Isani et al. (55),	Artemisinin and *A. annua* extract	D-17	/	Iron accumulation	/	Artemisinin and *A. annua* extract show a more potent cytotoxic activity on osteosarcoma cells possibly through the induction of ferroptosis

## Hopeful Investment in Osteosarcoma

Ferroptosis has been extensively studied in various disorders and exhibits great potential in antitumor therapy (12, 56). Tumor diseases marked by angiogenesis, which accounts for metastasis and poor prognosis, are in most cases treated with surgical resection, radiotherapy, or combined chemotherapy (57). Herein, the evocation of ferroptosis could be regarded as a new approach to halt osteosarcoma growth and could provide support for the treatment of difficult tumors (20, 58). Extensive research has identified several ferroptosis inducers for treating tumors, including erastin modulating system Xc^-^, sulfasalazine, sorafinib manipulating system Xc^-^, and RSL3 acting on GPX4 (40, 59). Previous studies on the role of ferroptosis in osteosarcoma mainly targeted GPX4 and reported encouraging results. Some tumor cell lines (possibly containing drug-resistant osteosarcomas) are also found to be resistant to ferroptosis burst when GPX4 is inhibited along with the acyl-CoA synthetase long-chain family member 4 (ACSL4) expression (18, 60). Therefore, induction of ferroptosis by blocking the FSP1/CoQ_10_ axis with concomitant GPX4 blockade may have positive effects on osteosarcoma treatment (13, 14). In addition, the level of FSP1 was found to be associated with tumor ferroptosis resistance, revealing that inhibitors of FSP1, earlier described as iFSP1, could be utilized to kill osteosarcoma cells, and the treatment could be further potentiated by the loss of GPX4 activity (14). Similarly, DHODH inhibitors are suggested to trigger ferroptosis through mitochondrial lipid peroxidation on those osteosarcoma cells with low GPX4 expression levels (17). However, abolishing the GCH1 network alone is not adequate to promote ferroptosis owing to CoQ_10_ interaction and the *de novo* biosynthesis of BH4 from GTP by GCH1 (46). In conclusion, targeting GPX4, FSP1, GCH1, and DHODH has a great potential for combating osteosarcoma.

## Discussion

Ferroptosis is a newly discovered form of regulated cell death with already identified four key regulators (61, 62). As a type of programmed cell death, ferroptosis induction offers an emerging strategy to treat tumors and other types of disorders. Although ferroptosis and its modulators, especially GPX4, have been extensively studied, much remains to be understood (12, 37). The mechanism of FSP1- and DHODH-mediated ferroptosis needs to be fully elucidated, and its regulators, inducers, or inhibitors are yet to be investigated. Apart from the aforementioned considerations, different cells may show diverse sensitivity toward ferroptosis or the possible FSP1 and DHODH inhibitors. Consequently, the exact dosage and method of delivering the possible FSP1 and DHODH inhibitors precisely are two major concerns that could limit the clinical application.

FSP1-mediated ferroptosis is also involved in inflammatory processes, hinting that ferroptosis is capable of recruiting immune cells in the tumor microenvironment (22, 63). The interaction between osteosarcoma cells and antitumor drugs is complicated. These drugs could induce cell death in various ways. The inflammatory process is an important component involved in osteosarcoma growth and development as tumor immunity is accompanied by inflammatory reactions (64). Although cell death types are multifarious, several forms could be induced under the same situation. During the process of tumor cell death, inflammation could be induced and related inflammatory cytokines such as TNF-α, IL-1β, and IL-6 further cause immune cell infiltration, which further results in the elimination of dead cells. FSP1 is a bidirectional protein associated with apoptosis or ferroptosis (13, 14, 42, 43, 65). It was previously identified to induce apoptosis in a caspase-independent and p53-independent manner, while it also protects cells from ferroptosis. Therefore, FSP1 may be considered as a decision-maker for the mechanism of cell death. Overall, a high FSP1 expression level confers protection against ferroptosis while inducing cell death through apoptosis. This could explain why almost all normal cells have low FSP1 expression levels, and low FSP1 expression can avoid cell apoptosis. Ferroptosis is an inflammation-regulated necrosis contrary to apoptosis. It can trigger inflammation, which helps purge tumor cells. Targeting FSP1 to promote ferroptosis is an interesting and theoretical strategy in osteosarcoma therapy. A future direction can be set to study the synergistic effect of FSP1 inhibitors and immunotherapy in the treatment of tumors.

In conclusion, the concept of FSP1 and its fast growth have shed new light on antitumor therapies, drastically changing the landscape of tumor treatment. However, further research on the correlation of FSP1 with osteosarcoma growth and progression and the underlying mechanism of FSP1 in osteosarcoma pathogenesis is required. Further research will be crucial for developing ferroptosis-based therapeutic regimens (64).

## Author Contributions

SW and LC raised the idea for the article and critically revised the manuscript. CQ, TL, DL, and DYL performed the literature search and data analysis. CQ, TL, LC, and SW were the major contributors in the drafting of the work. All authors contributed to the article and approved the submitted version.

## Conflict of Interest

The authors declare that the research was conducted in the absence of any commercial or financial relationships that could be construed as a potential conflict of interest.

## Publisher’s Note

All claims expressed in this article are solely those of the authors and do not necessarily represent those of their affiliated organizations, or those of the publisher, the editors and the reviewers. Any product that may be evaluated in this article, or claim that may be made by its manufacturer, is not guaranteed or endorsed by the publisher.

## References

[1] Yang J Zhang W. New Molecular Insights Into Osteosarcoma Targeted Therapy. Curr Opin Oncol (2013) 25(4):398–406. doi: 10.1097/CCO.0b013e3283622c1b 23666471

[2] Ottaviani G Jaffe N. The Epidemiology of Osteosarcoma. Cancer Treat Res (2009) 152:3–13. doi: 10.1007/978-1-4419-0284-9_1 20213383

[3] Gill J Gorlick R. Advancing Therapy for Osteosarcoma. Nat Rev Clin Oncol (2021) 18(10):609–24. doi: 10.1038/s41571-021-00519-8 34131316

[4] Moriarity BS Otto GM Rahrmann EP Rathe SK Wolf NK Weg MT. A Sleeping Beauty Forward Genetic Screen Identifies New Genes and Pathways Driving Osteosarcoma Development and Metastasis. Nat Genet (2015) 47(6):615–24. doi: 10.1038/ng.3293 PMC4767150 25961939

[5] Faisham WI Mat Saad AZ Alsaigh LN Nor Azman MZ Kamarul Imran M Biswal BM. Prognostic Factors and Survival Rate of Osteosarcoma: A Single-Institution Study. Asian J Clin Oncol (2017) 13(2):e104–e10. doi: 10.1111/ajco.12346 25870979

[6] Meyers PA Schwartz CL Krailo M Kleinerman ES Betcher D Bernstein ML. Osteosarcoma: A Randomized, Prospective Trial of the Addition of Ifosfamide and/or Muramyl Tripeptide to Cisplatin, Doxorubicin, and High-Dose Methotrexate. J Clin Oncol Off J Am Soc Clin Oncol (2005) 23(9):2004–11. doi: 10.1200/jco.2005.06.031 15774791

[7] Isakoff MS Bielack SS Meltzer P Gorlick R. Osteosarcoma: Current Treatment and a Collaborative Pathway to Success. J Clin Oncol Off J Am Soc Clin Oncol (2015) 33(27):3029–35. doi: 10.1200/jco.2014.59.4895 PMC4979196 26304877

[8] Green DR. The Coming Decade of Cell Death Research: Five Riddles. Cell (2019) 177(5):1094–107. doi: 10.1016/j.cell.2019.04.024 PMC6534278 31100266

[9] Green DR Evan Gi. A Matter of Life and Death. Cancer Cell (2002) 1(1):19–30. doi: 10.1016/s1535-6108(02)00024-7 12086884

[10] Vanden Berghe T Linkermann A Jouan-Lanhouet S Walczak H Vandenabeele P. Regulated Necrosis: The Expanding Network of non-Apoptotic Cell Death Pathways. Nat Rev Mol Cell Biol (2014) 15(2):135–47. doi: 10.1038/nrm3737 24452471

[11] Dixon SJ Lemberg KM Lamprecht MR Skouta R Zaitsev EM Gleason CE. Ferroptosis: An Iron-Dependent Form of Nonapoptotic Cell Death. Cell (2012) 149(5):1060–72. doi: 10.1016/j.cell.2012.03.042 PMC3367386 22632970

[12] Yang WS SriRamaratnam R Welsch ME Shimada K Skouta R Viswanathan VS. Regulation of Ferroptotic Cancer Cell Death by GPX4. Cell (2014) 156(1-2):317–31. doi: 10.1016/j.cell.2013.12.010 PMC4076414 24439385

[13] Doll S Freitas FP Shah R Aldrovandi M da Silva MC Ingold I. FSP1 is a Glutathione-Independent Ferroptosis Suppressor. Nature (2019) 575(7784):693–8. doi: 10.1038/s41586-019-1707-0 31634899

[14] Bersuker K Hendricks JM Li Z Magtanong L Ford B Tang PH. The CoQ Oxidoreductase FSP1 Acts Parallel to GPX4 to Inhibit Ferroptosis. Nature (2019) 575(7784):688–92. doi: 10.1038/s41586-019-1705-2 PMC6883167 31634900

[15] Kraft VAN Bezjian CT Pfeiffer S Ringelstetter L Müller C Zandkarimi F. GTP Cyclohydrolase 1/Tetrahydrobiopterin Counteract Ferroptosis Through Lipid Remodeling. ACS Cent Sci (2020) 6(1):41–53. doi: 10.1021/acscentsci.9b01063 31989025 PMC6978838

[16] Soula M Weber RA Zilka O Alwaseem H La K Yen F. Metabolic Determinants of Cancer Cell Sensitivity to Canonical Ferroptosis Inducers. Nat Chem Biol (2020) 16(12):1351–60. doi: 10.1038/s41589-020-0613-y PMC8299533 32778843

[17] Mao C Liu X Zhang Y Lei G Yan Y Lee H. DHODH-Mediated Ferroptosis Defence is a Targetable Vulnerability in Cancer. Nature (2021) 593(7860):586–90. doi: 10.1038/s41586-021-03539-7 PMC8895686 33981038

[18] Doll S Proneth B Tyurina YY Panzilius E Kobayashi S Ingold I. ACSL4 Dictates Ferroptosis Sensitivity by Shaping Cellular Lipid Composition. Nat Chem Biol (2017) 13(1):91–8. doi: 10.1038/nchembio.2239 PMC5610546 27842070

[19] Lee H Zandkarimi F Zhang Y Meena JK Kim J Zhuang L. Energy-Stress-Mediated AMPK Activation Inhibits Ferroptosis. Nat Cell Biol (2020) 22(2):225–34. doi: 10.1038/s41556-020-0461-8 PMC7008777 32029897

[20] Wu J Minikes AM Gao M Bian H Li Y Stockwell BR. Intercellular Interaction Dictates Cancer Cell Ferroptosis *via* NF2-YAP Signalling. Nature (2019) 572(7769):402–6. doi: 10.1038/s41586-019-1426-6 PMC6697195 31341276

[21] Yan B Ai Y Sun Q Ma Y Cao Y Wang J. Membrane Damage During Ferroptosis Is Caused by Oxidation of Phospholipids Catalyzed by the Oxidoreductases POR and CYB5R1. Mol Cell (2021) 81(2):355–69.e10. doi: 10.1016/j.molcel.2020.11.024 33321093

[22] Zheng J Conrad M. The Metabolic Underpinnings of Ferroptosis. Cell Metab (2020) 32(6):920–37. doi: 10.1016/j.cmet.2020.10.011 33217331

[23] Friedmann Angeli JP Schneider M Proneth B Tyurina YY Tyurin VA Hammond VJ. Inactivation of the Ferroptosis Regulator Gpx4 Triggers Acute Renal Failure in Mice. Nat Cell Biol (2014) 16(12):1180–91. doi: 10.1038/ncb3064 PMC4894846 25402683

[24] Alim I Caulfield JT Chen Y Swarup V Geschwind DH Ivanova E. Selenium Drives a Transcriptional Adaptive Program to Block Ferroptosis and Treat Stroke. Cell (2019) 177(5):1262–79.e25. doi: 10.1016/j.cell.2019.03.032 31056284

[25] Amaral EP Costa DL Mittereder L Mayer-Barber KD Andrade BB Namasivayam S. A Major Role for Ferroptosis in Mycobacterium Tuberculosis-Induced Cell Death and Tissue Necrosis. J Exp Med (2019) 216(3):556–70. doi: 10.1084/jem.20181776 PMC6400546 30787033

[26] Li Y Cao Y Xiao J Shang J Tan Q Ping F. Inhibitor of Apoptosis-Stimulating Protein of P53 Inhibits Ferroptosis and Alleviates Intestinal Ischemia/Reperfusion-Induced Acute Lung Injury. Cell Death Differ (2020) 27(9):2635–50. doi: 10.1038/s41418-020-0528-x PMC7429834 32203170

[27] Fang X Wang H Han D Xie E Yang X Wei J. Ferroptosis as a Target for Protection Against Cardiomyopathy. Proc Natl Acad Sci USA (2019) 116(7):2672–80. doi: 10.1073/pnas.1821022116 PMC6377499 30692261

[28] Fang X Cai Z Wang H Han D Cheng Q Zhang P. Loss of Cardiac Ferritin H Facilitates Cardiomyopathy *via* Slc7a11-Mediated Ferroptosis. Circ Res (2020) 127(4):486–501. doi: 10.1161/circresaha.120.316509 32349646

[29] Yao X Zhang Y Hao J Duan HQ Zhao CX Sun C. Deferoxamine Promotes Recovery of Traumatic Spinal Cord Injury by Inhibiting Ferroptosis. Neural Regen Res (2019) 14(3):532–41. doi: 10.4103/1673-5374.245480 PMC6334606 30539824

[30] Badgley MA Kremer DM Maurer HC DelGiorno KE Lee HJ Purohit V. Cysteine Depletion Induces Pancreatic Tumor Ferroptosis in Mice. Sci (New York NY) (2020) 368(6486):85–9. doi: 10.1126/science.aaw9872 PMC7681911 32241947

[31] Shen Z Song J Yung BC Zhou Z Wu A Chen X. Emerging Strategies of Cancer Therapy Based on Ferroptosis. Adv Mater (Deerfield Beach Fla) (2018) 30(12):e1704007. doi: 10.1002/adma.201704007 PMC6377162 29356212

[32] Hassannia B Vandenabeele P Vanden Berghe T. Targeting Ferroptosis to Iron Out Cancer. Cancer Cell (2019) 35(6):830–49. doi: 10.1016/j.ccell.2019.04.002 31105042

[33] Yee PP Wei Y Kim SY Lu T Chih SY Lawson C. Neutrophil-Induced Ferroptosis Promotes Tumor Necrosis in Glioblastoma Progression. Nat Commun (2020) 11(1):5424. doi: 10.1038/s41467-020-19193-y 33110073 PMC7591536

[34] Ubellacker JM Tasdogan A Ramesh V Shen B Mitchell EC Martin-Sandoval MS. Lymph Protects Metastasizing Melanoma Cells From Ferroptosis. Nature (2020) 585(7823):113–8. doi: 10.1038/s41586-020-2623-z PMC7484468 32814895

[35] Zou Y Henry WS Ricq EL Graham ET Phadnis VV Maretich P. Plasticity of Ether Lipids Promotes Ferroptosis Susceptibility and Evasion. Nature (2020) 585(7826):603–8. doi: 10.1038/s41586-020-2732-8 PMC8051864 32939090

[36] Friedmann Angeli JP Conrad M. Selenium and GPX4, a Vital Symbiosis. Free Radic Biol Med (2018) 127:153–9. doi: 10.1016/j.freeradbiomed.2018.03.001 29522794

[37] Ingold I Berndt C Schmitt S Doll S Poschmann G Buday K. Selenium Utilization by GPX4 Is Required to Prevent Hydroperoxide-Induced Ferroptosis. Cell (2018) 172(3):409–22.e21. doi: 10.1016/j.cell.2017.11.048 29290465

[38] Wang H An P Xie E Wu Q Fang X Gao H. Characterization of Ferroptosis in Murine Models of Hemochromatosis. Hepatol (Baltimore Md) (2017) 66(2):449–65. doi: 10.1002/hep.29117 PMC5573904 28195347

[39] Hadian K Stockwell BR. SnapShot: Ferroptosis. Cell (2020) 181(5):1188–.e1. doi: 10.1016/j.cell.2020.04.039 PMC8157339 32470402

[40] Xie Y Hou W Song X Yu Y Huang J Sun X. Ferroptosis: Process and Function. Cell Death Differ (2016) 23(3):369–79. doi: 10.1038/cdd.2015.158 PMC5072448 26794443

[41] Shen Z Liu T Li Y Lau J Yang Z Fan W. Fenton-Reaction-Acceleratable Magnetic Nanoparticles for Ferroptosis Therapy of Orthotopic Brain Tumors. ACS Nano (2018) 12(11):11355–65. doi: 10.1021/acsnano.8b06201 30375848

[42] Ohiro Y Garkavtsev I Kobayashi S Sreekumar KR Nantz R Higashikubo BT. A Novel P53-Inducible Apoptogenic Gene, PRG3, Encodes a Homologue of the Apoptosis-Inducing Factor (AIF). FEBS Lett (2002) 524(1-3):163–71. doi: 10.1016/s0014-5793(02)03049-1 12135761

[43] Wu M Xu LG Li X Zhai Z Shu HB. AMID, an Apoptosis-Inducing Factor-Homologous Mitochondrion-Associated Protein, Induces Caspase-Independent Apoptosis. J Biol Chem (2002) 277(28):25617–23. doi: 10.1074/jbc.M202285200 11980907

[44] Gong M Hay S Marshall KR Munro AW Scrutton NS. DNA Binding Suppresses Human AIF-M2 Activity and Provides a Connection Between Redox Chemistry, Reactive Oxygen Species, and Apoptosis. J Biol Chem (2007) 282(41):30331–40. doi: 10.1074/jbc.M703713200 17711848

[45] Cronin SJF Seehus C Weidinger A Talbot S Reissig S Seifert M. The Metabolite BH4 Controls T Cell Proliferation in Autoimmunity and Cancer. Nature (2018) 563(7732):564–8. doi: 10.1038/s41586-018-0701-2 PMC6438708 30405245

[46] Thöny B Auerbach G Blau N. Tetrahydrobiopterin Biosynthesis, Regeneration and Functions. Biochem J (2000) 347 Pt 1(Pt 1):1–16. doi: 10.1042/bj3470001 10727395 PMC1220924

[47] Garcia-Bermudez J Birsoy K. A Mitochondrial Gatekeeper That Helps Cells Escape Death by Ferroptosis. Nature (2021) 593(7860):514–5. doi: 10.1038/d41586-021-01203-8 33981062

[48] Shi Y Gong M Deng Z Liu H Chang Y Yang Z. Tirapazamine Suppress Osteosarcoma Cells in Part Through SLC7A11 Mediated Ferroptosis. Biochem Biophys Res Commun (2021) 567:118–24. doi: 10.1016/j.bbrc.2021.06.036 34147710

[49] Chen M Jiang Y Sun Y. KDM4A-Mediated Histone Demethylation of SLC7A11 Inhibits Cell Ferroptosis in Osteosarcoma. Biochem Biophys Res Commun (2021) 550:77–83. doi: 10.1016/j.bbrc.2021.02.137 33689883

[50] Lin H Chen X Zhang C Yang T Deng Z Song Y. EF24 Induces Ferroptosis in Osteosarcoma Cells Through HMOX1. Biomed Pharmacotherapy = Biomed Pharmacother (2021) 136:111202. doi: 10.1016/j.biopha.2020.111202 33453607

[51] Fu J Li T Yang Y Jiang L Wang W Fu L. Activatable Nanomedicine for Overcoming Hypoxia-Induced Resistance to Chemotherapy and Inhibiting Tumor Growth by Inducing Collaborative Apoptosis and Ferroptosis in Solid Tumors. Biomaterials (2021) 268:120537. doi: 10.1016/j.biomaterials.2020.120537 33260096

[52] Lv H Zhen C Liu J Shang P. β-Phenethyl Isothiocyanate Induces Cell Death in Human Osteosarcoma Through Altering Iron Metabolism, Disturbing the Redox Balance, and Activating the MAPK Signaling Pathway. Oxid Med Cell Longevity (2020) 2020:5021983. doi: 10.1155/2020/5021983 PMC7160723 32322335

[53] Lv HH Zhen CX Liu JY Shang P. PEITC Triggers Multiple Forms of Cell Death by GSH-Iron-ROS Regulation in K7M2 Murine Osteosarcoma Cells. Acta Pharmacol Sin (2020) 41(8):1119–32. doi: 10.1038/s41401-020-0376-8 PMC7468252 32132657

[54] Liu Q Wang K. The Induction of Ferroptosis by Impairing STAT3/Nrf2/GPx4 Signaling Enhances the Sensitivity of Osteosarcoma Cells to Cisplatin. Cell Biol Int (2019) 43(11):1245–56. doi: 10.1002/cbin.11121 30811078

[55] Isani G Bertocchi M Andreani G Farruggia G Cappadone C Salaroli R. Cytotoxic Effects of Artemisia Annua L. And Pure Artemisinin on the D-17 Canine Osteosarcoma Cell Line. Oxid Med Cell Longev (2019) 2019:1615758. doi: 10.1155/2019/1615758 31354901 PMC6637696

[56] Jiang L Kon N Li T Wang SJ Su T Hibshoosh H. Ferroptosis as a P53-Mediated Activity During Tumour Suppression. Nature (2015) 520(7545):57–62. doi: 10.1038/nature14344 25799988 PMC4455927

[57] Li L Qiu C Hou M Wang X Huang C Zou J. Ferroptosis in Ovarian Cancer: A Novel Therapeutic Strategy. Front Oncol (2021) 11:665945. doi: 10.3389/fonc.2021.665945 33996593 PMC8117419

[58] Wang W Green M Choi JE Gijón M Kennedy PD Johnson JK. CD8(+) T Cells Regulate Tumour Ferroptosis During Cancer Immunotherapy. Nature (2019) 569(7755):270–4. doi: 10.1038/s41586-019-1170-y PMC6533917 31043744

[59] Liang C Zhang X Yang M Dong X. Recent Progress in Ferroptosis Inducers for Cancer Therapy. Adv Mater (Deerfield Beach Fla) (2019) 31(51):e1904197. doi: 10.1002/adma.201904197 31595562

[60] Hangauer MJ Viswanathan VS Ryan MJ Bole D Eaton JK Matov A. Drug-Tolerant Persister Cancer Cells Are Vulnerable to GPX4 Inhibition. Nature (2017) 551(7679):247–50. doi: 10.1038/nature24297 PMC5933935 29088702

[61] Jiang X Stockwell BR Conrad M. Ferroptosis: Mechanisms, Biology and Role in Disease. Nat Rev Mol Cell Biol (2021) 22(4):266–82. doi: 10.1038/s41580-020-00324-8 PMC8142022 33495651

[62] Chen X Kang R Kroemer G Tang D. Broadening Horizons: The Role of Ferroptosis in Cancer. Nat Rev Clin Oncol (2021) 18(5):280–96. doi: 10.1038/s41571-020-00462-0 33514910

[63] Xie Z Hou H Luo D An R Zhao Y Qiu C. ROS-Dependent Lipid Peroxidation and Reliant Antioxidant Ferroptosis-Suppressor-Protein 1 in Rheumatoid Arthritis: A Covert Clue for Potential Therapy. Inflammation (2021) 44(1):35–47. doi: 10.1007/s10753-020-01338-2 32920707

[64] Zhong Z Sanchez-Lopez E Karin M. Autophagy, Inflammation, and Immunity: A Troika Governing Cancer and Its Treatment. Cell (2016) 166(2):288–98. doi: 10.1016/j.cell.2016.05.051 PMC4947210 27419869

[65] Marshall KR Gong M Wodke L Lamb JH Jones DJ Farmer PB. The Human Apoptosis-Inducing Protein AMID is an Oxidoreductase With a Modified Flavin Cofactor and DNA Binding Activity. J Biol Chem (2005) 280(35):30735–40. doi: 10.1074/jbc.M414018200 15958387

